# Understanding the interactability of chikungunya virus proteins *via* molecular recognition feature analysis

**DOI:** 10.1039/c8ra04760j

**Published:** 2018-07-31

**Authors:** Ankur Singh, Ankur Kumar, Vladimir N. Uversky, Rajanish Giri

**Affiliations:** School of Basic Sciences, Indian Institute of Technology Mandi Mandi Himachal Pradesh 175005 India rajanishgiri@iitmandi.ac.in; Department of Molecular Medicine and Byrd Alzheimer's Research Institute, Morsani College of Medicine, University of South Florida 12901 Bruce B. Downs Blvd. MDC07 Tampa Florida 33612 USA vuversky@health.usf.edu; Laboratory of New Methods in Biology, Institute for Biological Instrumentation, Russian Academy of Sciences Pushchino Moscow Region Russia; BioX Centre, Indian Institute of Technology Mandi VPO Kamand 175005 India

## Abstract

The chikungunya virus (CHIKV) is an alphavirus that has an enveloped icosahedral capsid and is transmitted by *Aedes* sp. mosquitos. It contains four non-structural proteins, namely nsP1, nsP2, nsP3, and nsP4, encoded at the 5′ end of the genome, and five structural proteins encoded at the 3′ end of the genome, including three glycosylated proteins, namely E1, E2, E3, a small 64 amino-acids glycoprotein 6K, and one non-glycosylated nucleocapsid protein C. The surface of this positive-stranded RNA alphavirus is covered with 80 trimeric glycoprotein spikes, which facilitate viral access into the host cell, with each consisting of three copies of E1-E2 heterodimers. The proper folding of p62, which is the precursor of E2, and formation of the E1-p62 heterodimers are controlled by E3, which is therefore essential for producing mature spikes on the alphavirus surface. Finally, 6K, a small 64 amino-acids glycoprotein, assists in the translocation of structural polyproteins to the endoplasmic reticulum and in the cleavage of p62 into mature structural proteins E2. The CHIKV proteins have been shown to contain variable levels of intrinsic disorder, often containing intrinsically disordered protein regions (IDPRs). IDPRs can interact with many unrelated partners, and these interactions are frequently accompanied by a transition from a disordered to ordered state. The corresponding sub-regions of IDPRs are acknowledged as molecular recognition features (MoRFs). Although the existence of IDPRs in CHIKV proteome has been analyzed, the prevalence of disorder-based protein–protein interactions (*i.e.* MoRF) in this virus have not been evaluated as of yet. To fill this gap, in our study, we utilized several computational methods to identify the MoRFs regions in CHIKV proteins. These computational tools included ANCHOR, DISOPRED3, MoRFpred and MoRFchibi_web server. These analyses revealed the presence of numerous MoRF regions in all the CHIKV proteins. In future, the results of this study could be used to identify the nature of chikungunya virus pathogenesis and might be helpful in designing drugs against this virus.

## Abstract 2

The chikungunya virus (CHIKV) is an alphavirus that has an enveloped icosahedral capsid and is transmitted by *Aedes* sp. mosquitos.

## Graphical abstract

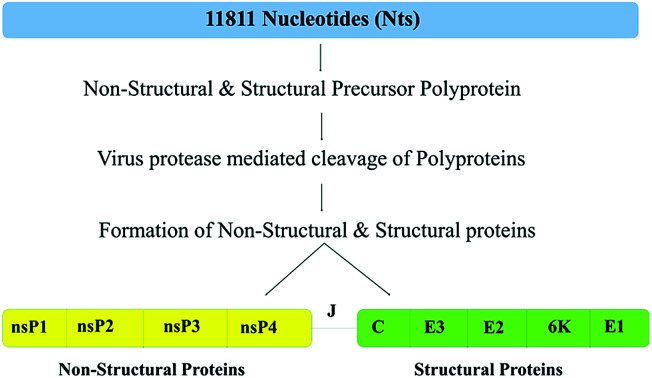

## Introduction

The chikungunya virus (CHIKV) is a mosquito-borne virus, first reported in Tanzania in 1952,^1–3^ and it later appeared as an epidemic in the French Reunion Island in 2005.^4^ By 2014, more than a million CHIKV infections were reported in the Americas alone, and epidemics were spread to more than 40 countries across Asia, Africa and Europe.^5^ CHIKV is transmitted to humans by *Aedes aegypti* and *A. albopictus* mosquitos. The symptoms of chikungunya disease are high fever, severe arthritis, myalgia and, rash.^6^ CHIKV belongs to the *Alphavirus* genus of the Togaviridae family, which is also associated with some other alphaviruses, such as the *Sindbis virus* (SINV), Semliki Forest virus (SFV), Ross River virus (RRV) and Venezuelan Equine Encephalitis virus (VEEV).^7^ CHIKV has a spherically shaped, *T* = 4 quasi-icosahedral symmetry with a ∼700 Å diameter capsid encapsulating a ∼11.8 kb long single-stranded, positive-sense RNA genome that encodes four non-structural (nsP1-4) and five structural proteins {capsid protein (CP), E3, E2, E1 and 6K},^8^ possessing the genomic organization 5′UTR-nsP1-nsP2-nsP3-nsP4-J-CP-E3-E2-6K-E1-polyA-3′UTR.^9^

**Fig. 1:**
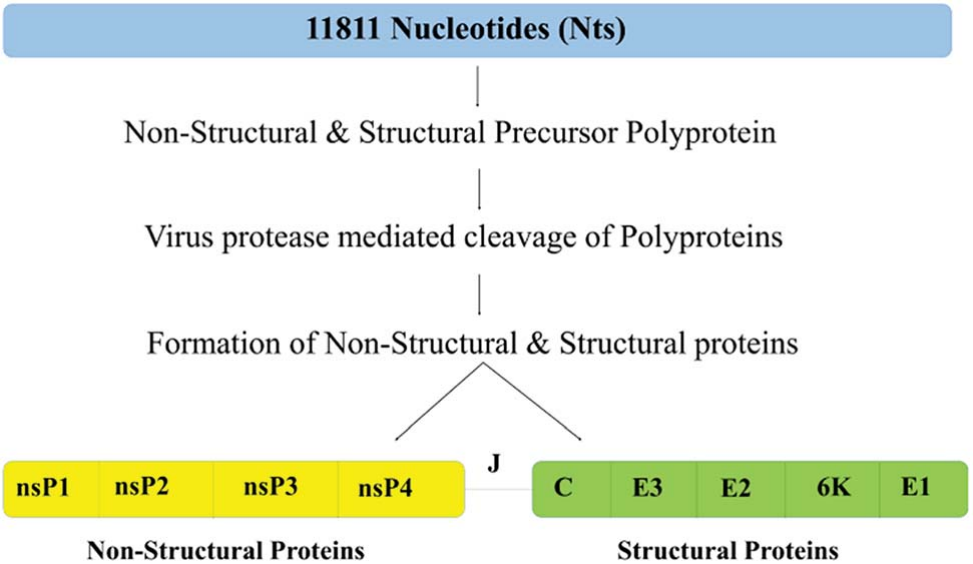
Schematic depiction of all the structural and non-structural proteins within the CHIKV polyprotein (UniProt ID: Q8JUX6 and Q8JUX5). CHIKV RNA 11811 bases (top bar, blue colour), translates into non-structural and structural precursor polyproteins of 2474 and 1244 residues, respectively, and after maturation by protease cleavage, it gives 4 non-structural proteins (left bar, yellow colour) and 5 structural proteins (right bar, green colour).

The CHIKV genome is characterized by the presence of two open reading frames (ORFs), ORF1 and ORF2, containing 7422 and 3744 nucleotides, encoding non-structural and structural proteins, respectively are connected by the junction region (J) that is used as a promoter for subgenomic RNA synthesis. Both, non-structural and structural proteins are synthesized as precursor polyproteins. Fig. 1 schematically represents the organization of the CHIKV proteome. The ORF1 encoded non-structural precursor polyprotein contains four proteins, namely nsP1 (535 amino acids, involved in capping and GTPase activity), nsP2 (798 amino acids, shows 5′ RTPase, helicase and protease activity), nsP3 (530 amino acids, has replicase activity and is involved in RNA synthesis) and nsP4 (611 amino acids, has RNA-dependent RNA polymerase activity).^10^

The ORF2-encoded structural precursor polyprotein includes five proteins, namely capsid protein CP (261 amino acids, involved in growth and assembly), envelope glycoproteins E1 (439 amino acids, facilitate membrane fusion), E2 (423 amino acids, helps in receptor binding), E3 (64 amino acids, controls the proper folding of the precursor of E2 (p62), regulates formation of the E1-p62 heterodimers, and protects the E2-E1 heterodimer from premature fusion with cellular membrane) and 6K (61 amino acids, assists in the translocation of structural polyproteins to the endoplasmic reticulum and cleavage of p62 into mature structural proteins E2).^10^

The structure of CHIKV was determined by cryo-electron microscopy (PDB ID: 3J2W).^11^ Furthermore, structural information is available for the protease domain of nsP2 (PDB ID: 3TRK), the macro-domain of nsP3 (PDB ID: 3GPG) and the mature envelope glycoprotein complex (complex of E1, E2 and E3; PDB ID: 3N41). We recently showed that the structural and non-structural proteins of CHIKV abundantly contain intrinsically disordered protein regions (IDPRs).^12^ This observation was in line with an important fact that a very noticeable part of any given proteome can be considered as “dark” since it includes proteins that are not amenable for structure determination by conventional methods, such as X-ray crystallography and electron microscopy.^13–17^ A large portion of the dark proteome is occupied by intrinsically disordered proteins (IDPs) of hybrid proteins containing ordered domains and IDPRs. Although IDPs/IDPRs are not folded into unique 3D structures, they have specific biological functions.^18–28^ These proteins exist as highly dynamic conformational ensembles and can attain highly diverse conformations, such as random coils, molten globules, pre-molten globules and flexible linkers.^29–31^ It is recognized now that the functional diversity of IDPs/IDPRs can be related to (or originate from) their extreme structural heterogeneity.^30,32,33^ Here, a structure of a protein molecule represents a mosaic of differently (dis)ordered segments, such as foldons (spontaneously foldable regions), non-foldons (regions that do not fold), semi-foldons (semi-folded regions), inducible foldons (regions that can at least partially fold at interaction with binding partner(s)) and unfoldons (regions that need to undergo functional unfolding to make a protein active).^30,32,33^ In addition to this mosaic structure, where different parts of a protein molecule are (dis)ordered to different degrees, the distribution of foldons, non-foldons, inducible foldons, semi-foldons and unfoldons is not steady but constantly changes over time. As a result, the protein structure is not crystal-like but is always morphing over time, with a given protein segment being able to have different structures at different time points.^30,32^ Because of the natural abundance, multitude of biological functions, and important regulatory roles of IDPs/IDPRs in various biological processes, unsatisfactory behaviors of many IDPs/IDPRs are commonly associated with various human maladies.^34–40^ As a result, IDPRs and IDPs serve as new and attractive targets for drug design.^41–44^

Though IDPs/IDPRs are functionally important for cell regulatory processes, their exact mechanistic functions are yet to be discovered.^45,46^ Functions of IDPs and IDPRs complement the functionality of ordered proteins and domains.^18,19,24–27,47–61^ For example, IDPs/IDPRs are known as promiscuous binders that can be involved in numerous interactions with many unrelated partners. IDPs/IDPRs also play vital roles in the establishment of several macromolecular complexes,^62^ with many IDPs/IDPRs showing disorder-to-order transition after binding to their partners.^63–65^ This mechanism is explained in great detail in the case of the interaction of C-myb protein with CREB-binding protein.^46,66,67^ IDPRs frequently contain molecular recognition features (MoRFs), which are relatively short (10–70 residues, loosely structured) sub-regions of IDPRs that endure disorder-to-order transition while interacting with particular binding partners.^64,65,68^ These MoRF regions play a crucial role in protein–protein interactions, metal binding and in cellular communications.^68–70^ MoRFs are divided into four groups based on their secondary structure in the bound state. MoRFs which form α-helix are called α-MoRFs, β-strands are formed by β-MoRFs, ι-MoRFs adopt an irregular structure during the interaction, whereas complex MoRFs form two or more types of secondary structures while binding to their partners.^68–70^ Amino acid residues present at the interface of MoRFs are relatively different from the residues present on the rest of the surface, with MoRFs typically possessing higher numbers of hydrophilic amino acids and prolines.^69^

The previous disorder analysis of the CHIKV proteome showed that nsP1, nsP3, nsP4, capsid and E3 proteins have IDPRs, involved in the maturation of viral particles and their replication.^12^ The earlier computational studies revealed that some flaviviruses,^71^ such as Dengue virus (DENV),^72^ hepatitis C virus (HCV),^73^ Zika virus (ZIKV), have a high prevalence of disorder and abundantly contain MoRF regions.^74,75^ The functional mechanism of MoRF regions in HCV was characterized by performing a yeast two-hybrid analysis assay.^76^

The fundamental focus of the present work was to analyze the presence of MoRFs in the CHIKV proteome. We believe that the analysis of the MoRF-based interactions of CHIKV proteins could represent an important platform for better understanding the molecular mechanisms of the pathogenicity of this virus because in some studies, it is reported that *Aedes aegypti* mosquitos show a co-infection with ZIKV and CHIKV without affecting its vector proficiency.^77,78^ In recent studies, it was found that the transmission of ZIKV & CHIKV also take place from mother to child during gestation and through breast milk, respectively, which could cause fatal infections in the infants.^79,80^ MoRF-based analysis of ZIKV has been completed and has shown the functional importance of the MoRF regions in its proteome.^75^ Hence, MoRF-centric analysis might represent a way to design drug molecules for the treatment of CHIKV infection.

## Materials and methods

In our previous study, we analyzed the occurrence of IDPRs in the CHIKV proteome, whereas the current study is dedicated to the analysis of the molecular recognition features (MoRFs) in the CHIKV proteome. The protein sequences used for the MoRF analysis were retrieved from the experimentally validated, reviewed UniProt database.^81^ These were the structural polyprotein (UniProt ID: Q8JUX5, 1248 residues) and non-structural polyprotein (UniProt ID: Q8JUX6, 2474 residues). The sequence of the CHIKV trans-frame protein was retrieved from the NCB NC_004162 entry. We used ANCHOR,^82^ MoRFpred,^83^ MoRFchibi_web^84–86^ and DISOPRED3 (ref. 87) to predict the MoRF regions in CHIKV proteins. Every predictor uses different sets of attributes for MoRF prediction. MoRFpred predicts MoRF regions mainly based on the fusion with sequence alignment-based annotations and the support vector machine (SVM).^83^ DISOPRED3 uses the SVM-RBF model and predicts MoRF regions based on data produced by an artificial neural network model.^87^ ANCHOR predicts disorder regions based on the biophysical characterization and expected energy calculations.^82^ MoRFchibi_web follows the Bayes rules to predict the MoRF regions,^84,85^ using two SVM models, SVMT and SVMSs, with various noise-tolerant kernels.^84,85^ The analyzed proteins and the results of the MoRF analysis are listed in Table 1.

**Table 1:** MoRF analysis of all CHIKV proteins by four different tools. Red numeric values show the MoRF regions that are common between two or more predictors

Name	Protein length (AA)	ANCHOR		MoRFchibi		MoRFpred		DISOPRED	
		Number of MoRFs ≥ 5 residues	MoRF-forming residues	Number of MoRFs ≥ 5 residues	MoRF-forming residues	Number of MoRFs ≥ 5 residues	MoRF-forming residues	Number of MoRFs ≥ 5 residues	MoRF-forming residues
nsP1	535	2	45–50, 507–515 507–515	3	40–52, 460–473, 506–513	3	45–49, 415–420, 504–512	None	None
nsp2	798	None	None	None	None	None	None	1	1–8
nsP3	530	6	305–311, 345–355, 382–396, 424–438, 466–483, 489–505	5	3–9, 35–41, 478–484, 493–506, 522–529	1	476–481	1	508–527
nsP4	611	1	79–88	2	1–8, 601–610	1	21–28	1	4–17
Capsid	261	7	1–16, 23–31, 36–59, 103–119, 121–134, 149–156, 223–230	2	1–27, 39–45	4	8–13, 46–51, 107–114, 219–224	2	62–87, 99–103
E1	439	None	None	None	None	1	230–234	2	422–426, 428–434
E2	423	3	97–104, 154–159, 259–264	None	None	1	10–15	None	None
E3	64	None	None	2	9–17, 33–64	None	None	None	None
6k	61	None	None	None	None	None	None	1	52–61
TF	76	None	None	1	51–76			None	None

Similar to the previous study, intrinsic disorder in the trans-frame protein was evaluated by a set of several per-residue disorder predictors, such as PONDR® VLXT,^88^ PONDR® VL3,^89^ PONDR® VSL2B^90^ and PONDR FIT.^91^ In these analyses, scores above 0.5 correspond to disordered residues/regions. PONDR® VLXT is not the most accurate predictor but has high sensitivity to local sequence peculiarities, which are often associated with disorder-based interaction sites;^88^ PONDR® VL3 possesses high accuracy in finding long disordered regions;^89^ PONDR® VSL2B is one of the most accurate stand-alone disorder predictors;^90^ whereas meta-predictor PONDR FIT is more accurate than each of its component predictors.^91^

## Results and discussion

Table 1 shows that the IDPRs involved in the disorder-based protein–protein interaction sites are present in every CHIKV protein (according to the results of at least one MoRF predictor utilized in this study). The largest number of MoRFs ranged from 1 to 7 in nsP2 and capsid proteins, respectively. Furthermore, in three multi-MoRF proteins, the location of several disorder-based protein–protein interaction sites was confirmed by at least two MoRF predictors.

### Analysis of the molecular recognition features in CHIKV non-structural proteins

#### Non-structural polyprotein and its processing

The non-structural CHIKV proteins are required for the viral RNA replication. CHIKV has four non-structural proteins: nsP1, nsP2, nsP3 and nsP4.^92^ These are synthesized as a short-lived nsP1234 polyprotein. The stages of maturation of these non-structural proteins are dependent on the infection stages. In fact, in the early infection stage, nsP1234 is cleaved *trans* by the nsP2 protease into the nsP123 and nsP4 proteins. Then, the nsP123 is cleaved *cis* into nsP1 and nsP23 by the nsP2 protease. Finally, due to the exposure of an ‘activator’ at the N-terminus of nsP23, nsP23 is cleaved into nsP2 and nsP3.^93^ The scenario of maturation is different at the late infection stage, where nsP1234 is quickly cleaved by nsP2 protease into nsP12 and nsP34 and then into the mature nsP1, nsP2, nsP3 and nsP4.^93^

#### Non-structural protein nsP1

**Fig. 2:**
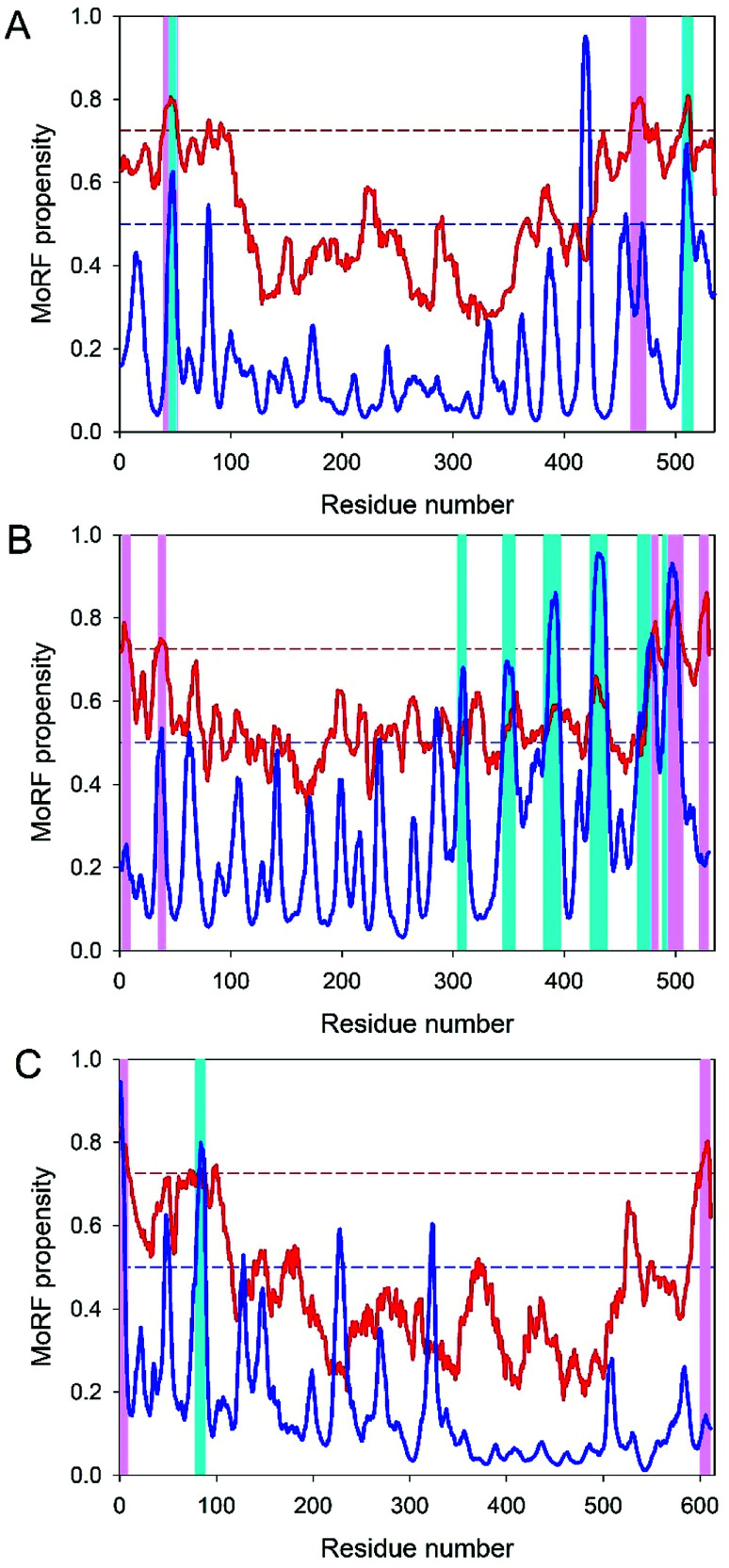
Evaluation of disorder-based interactivity of the CHIKV non-structural proteins nsP1 (A), nsP3 (B) and nsP4 (C). The presence of MoRF regions was evaluated by ANCHOR (blue lines) and MoRFchibi (red lines). The threshold for MoRF predictions by ANCHOR and MoRFchibi are 0.5 and 0.725. These thresholds are shown as dashed red and blue lines, respectively. Positions of MoRFs predicted by ANCHOR and MoRFchibi are shown by cyan and light pink bars, respectively. Note that for the nsP1 protein, positions of the C-terminal MoRF predicted by ANCHOR and MoRFchibi mostly overlap.

nsP1 is the first non-structural protein, and is a mRNA-capping enzyme of 535 residues that play various roles, such as 5′ capping, interaction of the replication complex with the host cytosolic membrane, downstream translation regulation and subgenomic RNA synthesis.^94^ It has two functional domains with the methyltransferase (MTase, residues 28-259) and nsP1 guanylyltransferase (GTase) activities. Our earlier analysis revealed that the average predicted per cent of intrinsic disorder (PPID) in nsP1 protein is 22.19%,^12^ with disorder being preferentially localized within the N- and C-terminal regions that are needed for capping and regulation of the downstream translation, respectively. Therefore, it is not surprising that MoRF regions are mainly present in the tails of nsP1. In fact, Fig. 2A represents the disorder profile generated for nsP1 by the MoRFchibi_web and ANCHOR servers. The results of these multi-tool analyses utilizing four different predictors of disorder-based binding sites are further summarized in Table 1, which clearly shows that this protein contains multiple MoRFs, each with the length of at least 5 residues. Importantly, regions 40–52 and 504–515 were predicted to contain MoRFs by three predictors (ANCHOR, MoRFchibi and MoRFPred), indicating the robustness of the MoRF predictions in this protein.

#### Non-structural protein nsP2

The nsP2 protease is the largest CHIKV protein and has almost 800 residues (residues 536–1333 of the non-structural polyprotein Q8JUX6). This viral protease contains three functional domains: the ^+^RNA virus helicase ATPase (residues 690–842), the ^+^RNA virus helicase C-terminal (residues 843–991) and the peptidase C9 (residues 1004–1327).^95^ The protease domain of CHIKV consists of two structural domains: a protease domain (residues ∼471–605) and a methyltransferase-like domain (residues 606–791), that function as a single unit and are both crucial for protease activity.^96^ In fact, nsP2 is the best studied non-structural protein of CHIKV, for which protease, NTPase, RNA triphosphatase and RNA helicase activities have been demonstrated.^96^ It also has a nucleolar localization signal (NoLS region, residues 1005–1024) and a nuclear localization signal (NLS motif, residues 1182–1186).^97^ With the lowest PPID of 5.92%,^12^ nsP2 is the most ordered CHIKV protein that does not have long IDPRs and contains only one MoRF (see Table 1) that could play some role in the complex functionality of the nsP2 protein.

#### Non-structural protein nsP3

nsP3 is the third non-structural protein of CHIKV. This 530 residue-long protein is characterized by a high disorder content (PPID = 42.12%), with disorder preferentially located within its C-terminal half.^12^ nsP3 contains three functional domains: N-terminal macro-domain, zinc-binding domain and variable C-terminal domain. The macro-domain, the crystal structure of which is known (PDB ID: 3GPG),^98^ has the capability to hydrolyze the ADP-ribose-1-phosphate, thereby acting as di-phosphoribose 1′′-phosphate phosphatase. Besides interaction with ADP-ribose, it can also bind, in a sequence-unspecific manner, to long, negatively charged polymers, such as DNA, poly(ADP-ribose) and RNA.^98^ The small zinc-binding domain that follows the macro domain has four conserved cysteine residues (Cys_263_, Cys_265_, Cys_288_ and Cys_306_). This zinc-binding domain is crucial for virus replication.^99^ The crystal structure was solved for the central part of the unprocessed nsP23 protein from *Sindbis virus* (SINV), which included protease and MT-like domains of nsP2 and macro and zinc-binding domains of nsP3, encompassing amino acids 1011–1675 of the nsP1234 polyprotein.^99^ In this structure, ∼40 residues between the macro domain and zinc-binding domain are disordered and the zinc-binding domain has an antiparallel α-helical bundle, two parallel β-strands and a zinc-coordination site.^99^ Zinc coordinating Cys_263_ and Cys_265_ are positioned in the loop between the last two α-helices, whereas C_288_ and C_306_ are placed at the C-termini of the two parallel β-strands.^99^ Finally, the 205 residue-long C-terminal domain represents a flexible tail that delivers an attachment site for marker proteins and, in another alphavirus, the Semliki forest virus has numerous phosphorylation sites.^100,101^ Low sequence conservation among alphaviruses and the presence of multiple phosphorylation sites in the C-terminal domain of nsP3 are in line with its highly disordered nature. In fact, in CHIKV, the C-tail is characterized by a PPID of 52.12%.^12^ Not surprisingly, most of the several MoRF identified in this protein are preferentially localized in this C-terminal tail (see Table 1 and Fig. 2B). nsP3 from SINV was shown to form several complexes with various host proteins in the virus-infected mosquito cells.^98^ Among the identified binding partners of the nsP3 were Rasputin (which is an insect cell-specific homolog of the Ras GTPase-activating protein-binding protein 1, G3BP1, which serves as a regulated effector of stress granule assembly), the heat shock protein HSC70, and one of the 14-3-3 proteins.^102^ Similarly, in infected vertebrate BHK-21 cells, nsP3 was found in complexes containing G3BP1, G3BP2, HSC70, nuclease-sensitive element-binding protein 1 (YBX1) involved in the pre-mRNA alternative splicing regulation, high concentrations of dsRNAs, and even entire ribosomes.^102^ It was suggested that the interaction of nsP3 with G3BP1 and G3BP2 interferes with the formation of normal stress granules and this represents a means by which the alphaviruses modify cellular translation and redirect it to the synthesis of virus-specific proteins.^102^ There is a high probability that MoRFs found in the C-tail of nsP3 serve as binding sites for interacting with various host proteins. Curiously, nsP3 of the Semliki Forest virus is phosphorylated at multiple serine residues (Ser_320_, Ser_327_, Ser_332_, Ser_335_, Ser_356_, Ser_359_, Ser_362_ and Ser_367_)^101^ and several threonine residues (*e.g.* Thr_344_ and Thr_345_)^100^ and contains a heavily phosphorylated peptide Gly_338_-Lys_415_, carrying 7–12 phosphates distributed over the 13 potential phosphorylation sites.^69^ Since these phosphorylation sites are located in close proximity to MoRFs, it is likely that the MoRF-driven interactability of nsP3 is further regulated by phosphorylation. Therefore, the functionality of nsP3 and especially its ability to bind to different partners is regulated by the interplay between the MoRF regions present within the C-tail of this protein and numerous phosphorylation events taking place in this region.

#### Non-structural protein nsP4

nsP4 is the fourth and the last non-structural protein. This 611 residue-long protein serves as an integral part of the alphavirus replication complex. It acts as an RNA-dependent RNA polymerase (RdRp) and can moderate phosphorylation of the α-subunit of elF2α (eukaryotic translation initiation factor) at the Ser_51_ residue,^103^ and also has terminal adenylyltransferase activity being able to specifically catalyse the addition of adenine to the 3′ end of an acceptor RNA in the presence of divalent cations.^104^ nsP4 is also essential for the RNA synthesis and for maintenance and repair of the poly(A) tail, which is an important element needed for viral genome replication.^104^ Although nsp4 has a PPID of 19.67%, it contains a 100 residue-long N-terminal region, which is highly unstructured (has a PPID of 73.25%)^12^ and is necessary for the proper functioning of nsP4.^104^Table 1 and Fig. 2C show that the N-tail of nsP4 is predicted to have MoRFs that might help this protein to function properly.

### Analysis of the molecular recognition features in CHIKV structural proteins

#### Structural polyprotein and its processing

The chikungunya virus contains five structural proteins, namely the capsid protein (CP), E3, E2, E1 and 6K protein. These structural proteins are encoded by the second ORF (3744 nucleotide long), which is translated into the CHIKV structural precursor polyprotein of 1244 residues with the [CP-p62-6K-E1] organization, where p62 (or pE2) is an E2-E3 precursor polyprotein containing unprocessed E2 and E3 proteins. The structural precursor polyprotein [CP-p62-6K-E1] undergoes sequential maturation. First, CP is autocatalytically cleaved off the precursor and is then immediately used for the encapsidation of new plus-strand RNA molecules,^105^ whereas the envelope polyprotein precursor p62-6K-E1 is translocated to the endoplasmatic reticulum (ER). Here, this polyprotein is processed by the host signalases that cleave it at the N- and C-terminal ends of the 6K protein. This generates the p62 precursor, 6K and E1 proteins that are anchored to the ER membrane.^105^ This processing triggers the formation of the p62-E1 heterotrimers in the Golgi compartment. The formation of this heterodimeric p62-E1 complex is essential for the correct folding.^106^

The p62-E1 heterotrimers trimerize to form the viral ‘spikes’. Next, in the *trans*-Golgi system, p62 is cleaved, in a furin-dependent manner, into mature E2 and E3 glycoproteins.^101^ This maturation of p62 into E3 and E2 during transport to the cell surface primes the spikes for subsequent fusogenic activation for cell entry.^106^

#### Capsid protein

**Fig. 3:**
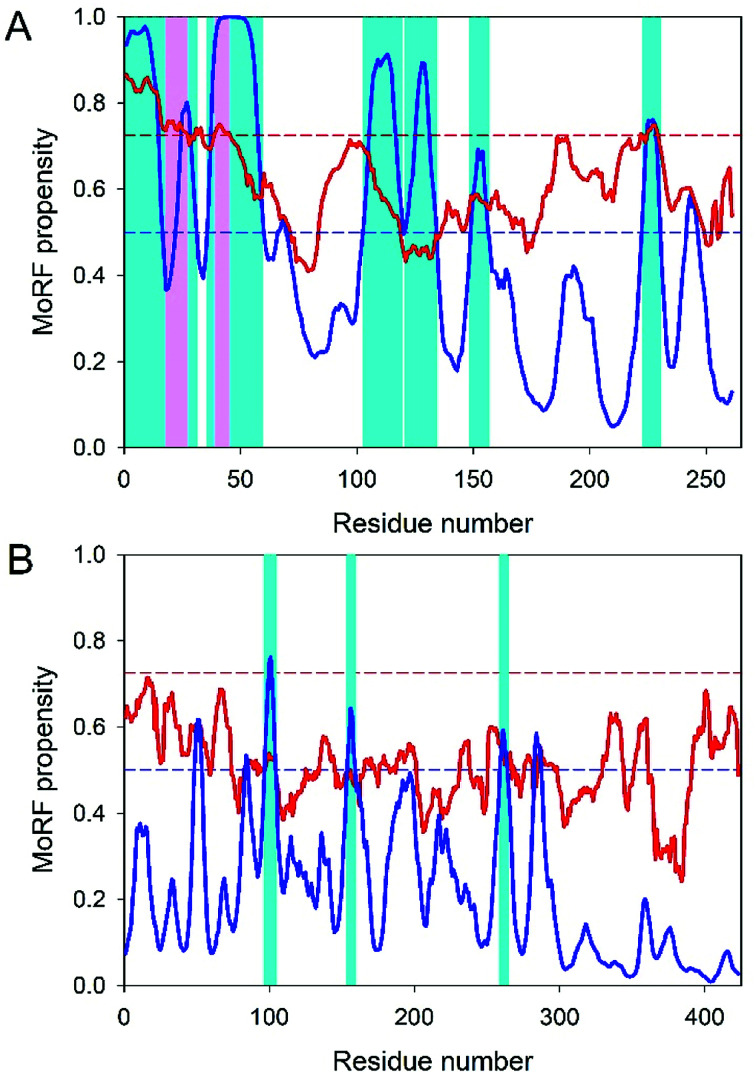
Evaluation of the disorder-based interactivity of the CHIKV structural proteins CP (A) and E2 (B). The presence of MoRF regions was evaluated by ANCHOR (blue lines) and MoRFchibi (red lines). The threshold for MoRF predictions by ANCHOR and MoRFchibi are 0.5 and 0.725. These thresholds are shown as dashed red and blue lines, respectively. Positions of MoRFs predicted by ANCHOR and MoRFchibi are shown by cyan and light pink bars, respectively. Note that for the CP, the positions of the N-terminal MoRFs predicted by ANCHOR and MoRFchibi mostly overlap.

CP is a 261 residue-long protein with the primary function of forming the nucleocapsid capable of self-cleavage from the structural polyprotein prior to the genomic RNA binding.^107^ CP was identified as a polyfunctional protein, which in addition to the nucleocapsid formation plays a number of crucial roles in the assembly and budding of alphaviruses as it able to inhibit and/or regulate viral replication and act as a pleiotropic regulator of synthesis of host and viral proteins.^108,109^ CP, being secreted out to the plasma membrane, is able to interact with the C-terminal region of E2 for the initiation of virion budding. This polyfunctionality is reflected in the domain organization of CP that has an RNA binding N-terminal domain (residues 1–113) and a 147 residue-long C-terminal protease domain. Although the overall PPID of CP is 46.35%. The N-terminal 110 residues of this protein are highly disordered, being characterized by a PPID of 96.13%.^12^ This N-terminal disordered domain is enriched in positively charged lysine and arginine residues, contains numerous proline residues, is involved in protein–protein interactions, has a ribosome binding site (residues 91–100) and is able to bind to the genomic RNA *via* the coiled-coil region (residues 81–105).^110^ It also includes two nuclear localization signals (residues 60–77 and 84–99) for the capsid protein in translocation to the host cell nucleus.^111^ The C-terminal domain of CP is highly-conserved among alphaviruses. It is a globular chymotrypsin-like serine protease containing a catalytic triad (His_139_, Asp_161_ and Ser_213_) responsible for the autoproteolytic activity by which CP is cleaved from the structural precursor polyprotein.^112^ The protease domain of CP also contains a binding site for the spike protein, which is a hydrophobic pocket near the CP protease substrate binding site that plays a role in binding of the E2 glycoprotein endodomain to capsid.^111^ Because of its highly disordered nature, CP possesses numerous MoRF regions related to the functionality of this protein. In fact, Fig. 3A represents the multi-MoRF profile generated for CP by ANCHOR, whereas Table 1 summarizes the results of the multi-tool analysis of the potential disorder-based interactability of this protein.

#### Spike glycoprotein E1

Glycoprotein E1 is a 439 residue-long type I transmembrane protein that serves as an important part of the spikes found on the CHIKV envelope. There are 80 such spikes in a CHIKV virion, each made from three E2-E1 heterodimers. Viral spikes mediate membrane fusion to deliver the viral genome into the host cell. Here, E1 glycoprotein plays a major role in virus–host cell membrane fusion undergoing a large conformational change.^113,114^

For its fusion activity, E1, which has structural domains I, II and III, possesses a fusion loop (residues 893–910 of structural polyprotein or residues 84–101 of the mature E1 glycoprotein) located at the tip of the Domain II. Virus–host cell fusion is triggered by the acidic endosomal environment that promotes E2-E1 heterodimer dissociation and rearrangement of the E1 into fusogenic homotrimers.^115,116^ E1 of CHIKV belongs to the type II fusion proteins that form trimers of hairpins composed of β-sheets in the postfusion state.^117–119^ Being isolated, the 18 residue-long fusion region of E1 is able to induce liposome fusions in a pH-independent manner.^120^ In the presence of dodecylphosphocholine (DPC) micelles, this peptide adopts β-type or extended conformations, with the aromatic side chains of Tyr_85_, Phe_87_, Tyr_93_ and Phe_95_ being well-packed in an aromatic core.^120^ With a mean PPID of 7.74%, E1 is the second most ordered protein in the CHIKV proteome.^12^ In agreement with this observation, Table 1 shows that E1 is predicted to have one (MoRFPred) or two (DISOPRED) MoRFs, suggesting that this protein is not engaged in extensive disorder-based interactions.

#### Spike glycoprotein E2

Glycoprotein E2 is derived from the CHIKV structural polyprotein in a form of the p62 (or pE2) precursor polyprotein containing unprocessed E2 and E3 proteins. p62 is another type I membrane protein that forms a heterodimeric p62-E1 complex, the trimerization of which leads to the formation of the viral ‘spikes’. This cotranslational formation of the p62-E1 complex is crucial for the correct folding of proteins. The p62-E1 complex undergoes glycosylation in ER and then undergoes maturation, *via* furin-driven cleavage in the *trans*-Golgi system, to generate mature E2 and E3 glycoproteins.^101^ Although in the E1-E2 complex, E1 is responsible for cell fusion, the primary function of E2 is receptor binding and cell entry.^118,121^ Each of these viral glycoproteins have a transmembrane helix traversing the lipid bilayer.^122^ Spike glycoprotein E2 is a 423 residue-long protein that belongs to the immunoglobulin superfamily and contains three structural domains (A, B and C).^105^ Domains A and B of the E2 protein form a groove that is used for the insertion of the fusion loop of the E1 protein (in a form of β-hairpin) at the formation of a heterodimer complex. Although E2 is characterized by a relatively low level of intrinsic disorder (its mean PPID is 15.19%),^12^ it is predicted to have several MoRF regions preferentially located in the N-terminal half of protein (see Table 1 and Fig. 3B). Curiously, spike protein E2 is N-glycosylated at residues Asn_263_ and Asn_273_,^123^ located within or in close proximity to one of the MoRFs, suggesting that the disorder-based binding activity of E2 can be further regulated by posttranslational modifications.

#### Envelope protein E3

A peripheral glycoprotein E3 is a short protein (it contains 64 residues), which is released from the p62 precursor protein during the furin-mediated maturation. E3 provides the signal sequence for p62 translocation into the ER.^124^ In the p62-E1 complex, E3 (which is located at the N-terminus of p62) protects the fusion loop of the immature virus.^118,121,123^ Therefore, E3 helps in the heterodimerization of E1-p62 and ensures that the E1 fusion loop is not involved in the premature fusogenic activation.^125,126^ The crucial role of E3 in the E1-E2 heterodimerization is supported by the observation that recombinant E2 lacking the E3 moiety does not dimerize with E1.^127^ Although the glycoprotein E3 does not interact with the fusion protein E1 directly, it makes contact with the domain B of E2, acting as a brace that keeps the specific orientation of the domain B relative to domain A to ensure creation of the aforementioned groove needed for the accommodation of the fusion loop.^106^ It was also established that in some alphaviruses, such as CHIKV, Semliki Forest and Venezuelan equine encephalitis viruses, E3 remains in contact with the mature spikes.^128,129^ It was shown that two tyrosine residues of E3, Tyr_47_ and Tyr_48_ play an important role in the stability of the E3-E2 heterodimer and, therefore, are related to the biogenesis of the CHIKV envelope protein.^123^ Our earlier disorder analysis indicated that the E3 glycoprotein is the most disordered protein in the CHIKV proteome (it has a PPID value of 61.72%).^12^Table 1 shows that E3 was predicted by MoRFchibi to have two MoRFs. Importantly, the C-terminally located MoRF (residues 33–64) includes the aforementioned Tyr_47_ and Tyr_48_ residues that are engaged in direct interaction with the E2 protein (together with Glu_39_ and Asp_40_, which form salt bridges with E2 Arg_251_),^106^ indicating that the E2-E3 complex formation can be driven by the intrinsic disorder-based interaction accompanied by the disorder-to-order transition in the E3 protein.

#### 6K protein

The 6K protein is a small (61 residues), hydrophobic, cysteine-rich, acylated protein with numerous functions, ranging from an involvement in envelope protein processing to membrane permeabilization, virus budding and virus assembly. In structural polyprotein, the 6K protein provides the cleavage sites for signalase. 6K is predicted to have two transmembrane (TM) regions with different functions: an N-terminally located TM region, which is potentially involved in the ion-channel activity, and a C-terminal TM, which mediates translocation of the E1 protein into ER.^130^ Furthermore, the N-terminal region preceding the transmembrane anchor contains two conserved interfacial sequence motifs (Tyr-Leu-Trp and Phe-Trp-Val) separated by Asn and Gln residues, which define the ability of the pre-transmembrane region to partition into the membrane interface and are important for virus budding and modification of the membrane permeability.^131^ Although 6K is synthesized in equal amounts relative to the other envelope proteins, only small amounts of 6K are actually incorporated into virions,^132^ with the ratio 6K to E1 + E2 ranging from 0.08 to 0.12.^133^ The CHIKV 6K protein is characterized by a mean PPID score of 11.88%,^12^ and was predicted by DISOPRED to have one C-terminally located MoRF (see Table 1).

#### Trans-frame protein

In the Semliki Forest virus and in other alphaviruses, the ribosomal −1 frameshift might take place at a conserved UUUUUUA motif within the sequence encoding 6K.^134^ An estimated efficiency of such a frameshifting event is approximately 10–18%. It results in the synthesis of an additional trans-frame (TF) protein that has a molecular mass of 8 kDa and contains a C-terminal extension in the −1 open reading frame (ORF).^134^ TF shares the N-terminal amino acid sequence, including the first TM region implicated in ion-channel activity, with 6K, but also contains a unique, basic C-terminus, which is ∼15 residues longer than that of 6K and is relatively conserved among the alphaviruses due to the presence of a conserved stop codon in the −1 ORF.^134^ Functional analysis of the TF protein revealed that it is important for alphavirus assembly and retains ion-channel activity analogous to that of 6K. On the other hand, envelope protein transit to the cell surface, genome replication and particle infectivity were not affected by the lack of the TF production.^135^

**Fig. 4:**
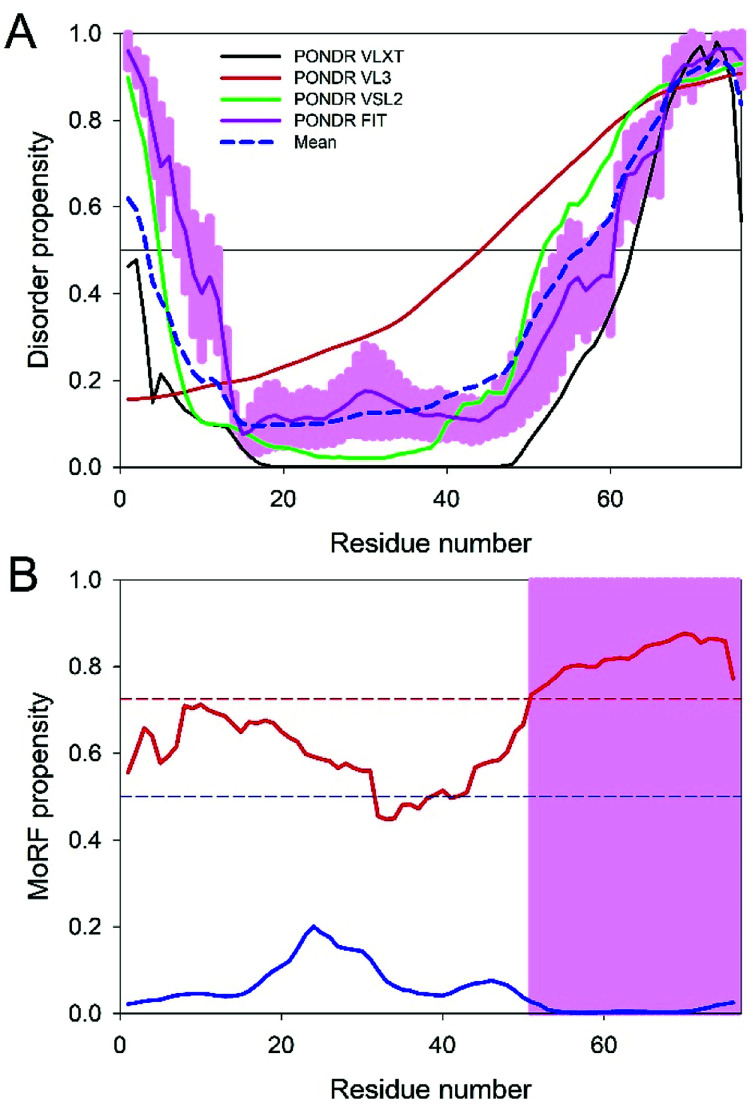
Evaluation of disorder predisposition (A) and disorder-based interactivity (B) of the CHIKV TF protein. Intrinsic disorder propensity was evaluated by four predictors of the PONDR family: PONDR® VLXT (black line), PONDR® VL3 (red line), PONDR® VSL2B (green line) and PONDR FIT (pink line). Light pink shadow around PONDR FIT curve shows the error distribution. Blue dashed line represents the mean disorder propensity calculated by averaging the outputs of individual predictors. The presence of MoRF regions was evaluated by ANCHOR (blue line) and MoRFchibi (red line). The threshold for MoRF predictions by ANCHOR and MoRFchibi are 0.5 and 0.725. These thresholds are shown as dashed red and blue lines, respectively. Positions of MoRFs predicted by ANCHOR and MoRFchibi are shown by cyan and light pink bars, respectively.

Since the disorder status of the CHIKV TF protein has not been evaluated as of yet, we conducted a multi-tool analysis of this protein using four predictors of the PONDR family. The results of this analysis are summarized in Fig. 4A. It can be clearly seen that the TF protein has a significant level of predicted disorder. In fact, its mean PPID (36.72%) noticeably exceeded the corresponding value obtained earlier for the CHIKV 6K protein (11.88%),^12^ indicating that the frameshift-generated extension of the C-terminal region of TF is highly disordered. Fig. 4B shows that this mostly disordered expansion can serve as a disorder-based interaction site. These observations suggest that the −1 frameshift within the sequence encoding 6K generates a protein with new functional capabilities.

## Conclusions

The pathogenic mechanism of an arthropod-borne CHIKV is not entirely understood as of yet because of the sparsity of currently available structural information about viral proteins. The goal of this study was to partially fill the gap by providing data on the prevalence of disordered based protein–protein interactions in the chikungunya proteome. Based on the multi-tool computational analysis it was concluded that all CHIKV proteins have at least one MoRF. Since most of the CHIKV proteins are involved in interactions with various host proteins, the discovery of the ample presence of MoRF regions in these proteins delivers some new insights into the molecular mechanisms of action of these proteins. It seems that the presence of disorder-based binding sites represents a characteristic feature of CHIKV proteins. We believe that, as described in this study, the insights into there being a multitude of disorder-based protein–protein interactions (MoRFs) in CHIKV proteins delivers a new angle needed for better understanding the molecular mechanisms involved in the CHIKV pathogenesis and viral infectivity. The disorder-based protein–protein interactions can provide a novel way to design specific drug molecules against this virus once the exact roles of all the CHIKV proteins are deciphered.

## Conflicts of interest

There are no conflicts to declare.

## Supplementary Material
